# Emerging roles of the ciliary-mitochondrial axis in cellular homeostasis and neuroprotection

**DOI:** 10.1186/s44477-025-00005-w

**Published:** 2025-11-25

**Authors:** Emiko Miller, Peter Bambakidis, Phoebe Templin, Bindu D. Paul, Andrew A. Pieper

**Affiliations:** 1 Department of Psychiatry, Case Western Reserve University, Cleveland, OH, USA; 2 Department of Neurosciences, Case Western Reserve University, Cleveland, OH, USA; 3 Geriatric Psychiatry, GRECC, Louis Stokes VA Medical Center, Cleveland, OH, USA; 4 Institute for Transformative Molecular Medicine, School of Medicine, Case Western Reserve University, Cleveland, OH, USA; 5 Brain Health Medicines Center, Harrington Discovery Institute, University Hospitals Cleveland Medical Center, Cleveland, OH, USA; 6 Department of Physiology, Pharmacology & Therapeutics, Johns Hopkins University School of Medicine, Baltimore, MD, USA; 7 Department of Psychiatry and Behavioral Sciences, Johns Hopkins University School of Medicine, Baltimore, MD, USA; 8 The Solomon H. Snyder Department of Neuroscience, Johns Hopkins University School of Medicine, Baltimore, MD, USA; 9 Lieber Institute for Brain Development, Baltimore, MD, USA; 10 Department of Pathology, Case Western Reserve University, Cleveland, OH, USA

**Keywords:** Primary cilia, Mitochondria, Neurodegeneration, Alzheimer’s disease, Ciliopathy

## Abstract

Primary cilia and mitochondria, long studied as separate cellular players, are now recognized as a tightly coupled signaling and metabolic hub whose interplay powerfully shapes cell fate. The bidirectional ciliary-mitochondrial axis integrates extracellular sensing, calcium dynamics, bioenergetics, and organelle quality control to drive adaptive responses to stress and sustain neuronal resilience. Recent studies reveal compelling associations that merit further investigation, such as the impact of primary cilium-initiated signaling cascades on mitochondrial dynamics and mitophagy, and the effects on cilia of shifting mitochondrial metabolic states. Dysfunction at any node in this axis has potential to trigger neurodegeneration. Framing primary cilia and mitochondria as a coordinated physiologic axis enables reconsideration of neurodegeneration and reveals novel, tractable entry points for therapeutic restoration of brain homeostasis. This review traces the field’s evolution, synthesizes key molecular mechanisms, and highlights exciting translational opportunities to harness the ciliary-mitochondrial axis for neuroprotection.

## Background: Introduction to the ciliary-mitochondrial axis

Most human cells contain mitochondria [1] and a single primary cilium [2, 3]. Primary cilia are sensory organelles that serve as an organized hub for signaling pathways [3], while mitochondria meet the cell’s energetic and metabolic needs [4]. Historically, these organelles have been studied separately, leaving cilia-mitochondrial interactions understudied until recently. Here, we highlight the emerging concept of a ciliary-mitochondrial axis.

Primary cilia were first observed in 1677 by Antoni van Leeuwenhoek, a pioneer in microscopy, as hair-like extensions (termed “little feet”) on protozoa [5], and later termed “cilia” (Latin for eyelash) by Otto Friedrich Müller [6]. Mitochondria, on the other hand, were not described until more than a century later. Albert von Kölliker observed intracellular threadlike structures in muscle fibers termed “sarcosomes” [7], Richard Altmann later renamed them as “bioblasts” [8], and Carl Benda ultimately coined the term “mitochondria” in 1898 [9]. With both organelles, structural identification preceded functional understanding. Initially, cilia were noted as solitary appendages on epithelial cells [10, 11], and mitochondria were considered granular or threadlike inclusions with uncertain roles [7].

From the late 19th to mid-twentieth century, functional insight into primary cilia lagged far behind the field’s understanding of mitochondrial function. Advances in light microscopy and iron-hematoxylin staining showed that most cells bear a single primary cilium [10], which Charles Joseph discovered to be conserved across diverse tissues [12]. Later, the introduction of electron microscopy in the 1950 s revealed the presence of non-motile cilia in neural tissue [13, 14]. However, cilia were still considered vestigial. By contrast, mitochondrial research accelerated during this time, with Peter Mitchell’s chemiosmotic theory explaining ATP generation via mitochondrial F_1_F_0_-ATP synthase [15], Margit and Sylvan Nass’s discovery of mitochondrial DNA [16], and Lynn Margulis’s conception of the endosymbiotic origin of mitochondria [17].

By the turn of the twenty-first century, however, both organelles had come to be appreciated as central regulators of physiology and disease. The discovery of intraflagellar transport by Rosenbaum in 1993 uncovered the molecular machinery of cilia [18], and Witman and Cole’s discovery of the link between ciliary defects and polycystic kidney disease established ciliopathies as a new disease class [19]. Subsequent work revealed ciliary roles for Hedgehog, Wnt, and other signaling pathways across development and adulthood [20], implicating ciliary dysfunction in aging and neurodegeneration [21]. In parallel, mitochondria were recognized as dynamic regulators of apoptosis [22], calcium signaling [23], redox balance [24], innate immunity [25], and aging [26].

Today, accumulating evidence supports a tightly coordinated, bidirectional interplay between primary cilia and mitochondria, which is mediated by shared signaling cascades and structural interactions. Here, we describe the currently understood details of this integrative system known as the “ciliary-mitochondrial axis.” Through coordinated regulation of energetics, mitochondrial quality control, calcium homeostasis, and stress responses, this axis influences cellular adaptation and neuronal survival. Importantly, dysfunction in one organelle can provoke reciprocal pathology in the other, creating a feedback loop with potential to initiate and propagate neurodegeneration. This interdependence highlights the ciliary-mitochondrial axis as a promising target for therapeutic intervention.

## Bidirectional regulation between primary cilia and mitochondria

### Spatial and structural coupling of primary cilia and mitochondria

**Fig. 1:**
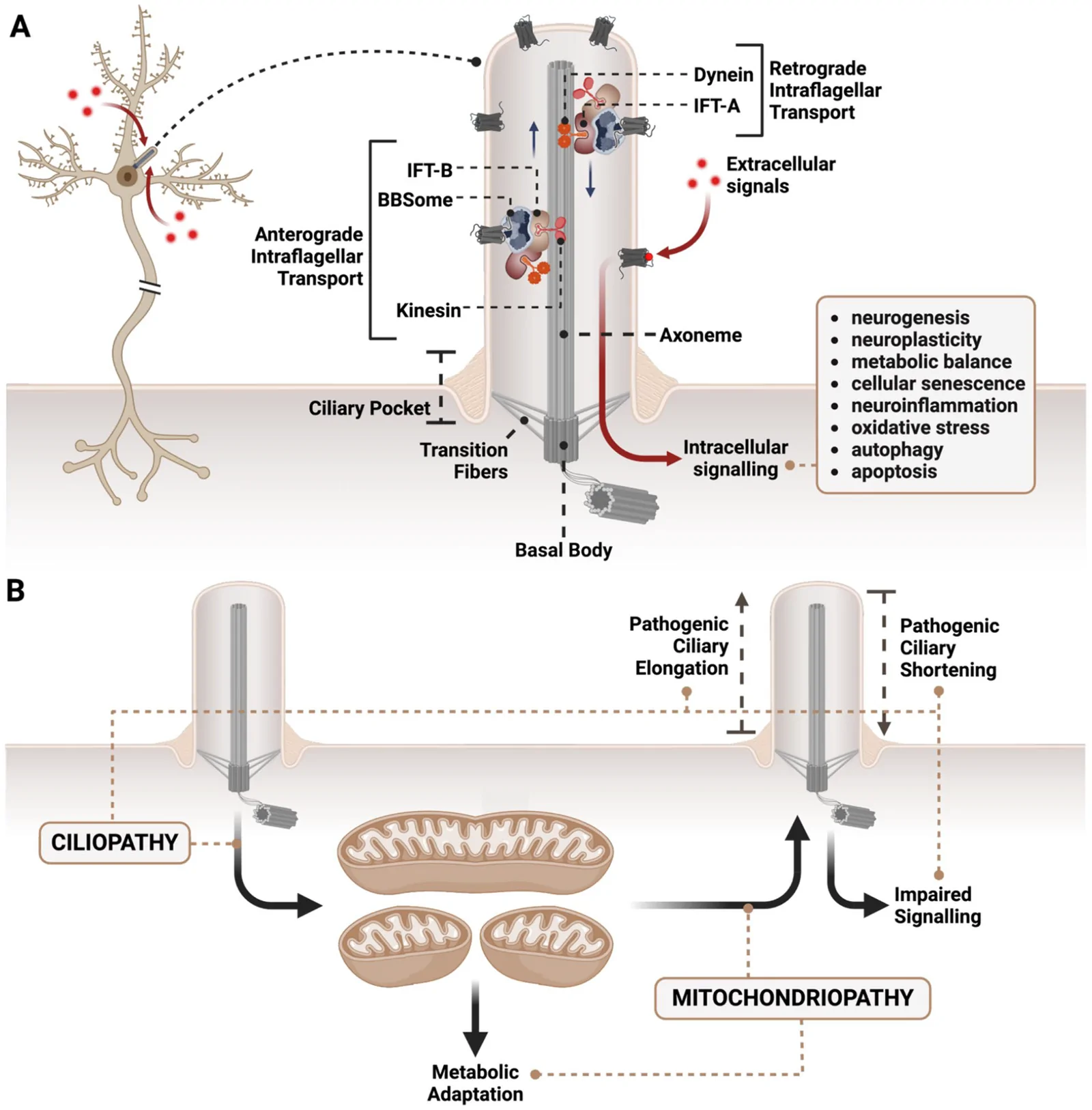
Interaction between primary cilia and mitochondria structure and signaling. **A** Basic architecture of primary cilia. All primary cilia share a basic architecture, including the basal body, a microtubule-based axoneme, and a ciliary membrane that is continuous with the cellular plasma membrane. The axoneme is composed of microtubules arranged in a highly conserved 9 + 0 pattern, which serve as “train-tracks” for intraflagellar transport (IFT) mediated by various IFT-A and IFT-B proteins. The basal body is derived from the mother centriole, which helps anchor the cilium to the cell body through transition fibers and subdistal appendages. The base of the cilium contains a diffusion barrier, meaning that structural proteins required to maintain the axoneme need to be delivered into the cilia, while other signaling proteins must be transported out of the cilia. This transport process is tightly regulated by the transition fibers. Once cargo enters the cytosolic compartment of the primary cilia, the IFT system bidirectionally transports it along the axoneme. The BBSome, loaded with membrane proteins, is transported by IFT complexes along the microtubules via the motor proteins dynein and kinesin. The IFT-B protein complex transports cargo anterogradely towards the ciliary tip, while the IFT-A protein complex mediates retrograde transport back to the ciliary base. The primary cilium’s compact cytosolic space and specialized structure make it an ideal hub for sensory function and signaling. Despite its size, the primary cilium houses a highly organized and specialized collection of receptors, ion channels, and signaling molecules that coordinate rapid intracellular responses to extracellular signals. Functionally, this allows primary cilia to transduce mechanical and chemical signals across various cell types. **B** Interdependence between primary cilia and mitochondria in cellular homeostasis. Mitochondrial dysfunction can disrupt cilia health, manifesting as pathogenic ciliary elongation or shortening, which are hallmarks of ciliopathy. Conversely, defective cilia signaling harms mitochondrial health, exacerbating mitochondrial dysfunction. This bidirectional regulation underscores the role of cilia-mitochondria crosstalk in maintaining cellular function and its dysregulation in disease states

Primary cilia arise from the centrioles and typically extend a single extracellular microtubule-based projection with membrane-localized receptors (for example, G protein coupled receptors (GPCRs)) that detect chemical and mechanical cues [3] (Fig. 1). Cilia lack protein-synthesizing machinery and thus rely on highly conserved and specialized transport systems, including intraflagellar transport (IFT) complexes and the BBSome, to traffic ciliary proteins [27]. Because primary cilia are very narrow (approximately 250 nm) and mitochondria are substantially larger (approximately 0.5–1.5 μm), mitochondria cannot enter cilia. Instead, mitochondria are frequently clustered near the ciliary base in many cell types, including fibroblasts [28, 29], epithelial cells [30], and rod photoreceptors [31]. Evidence for this localization in neurons is more limited, but 3D ultrastructural studies report the presence of mitochondria near centrioles in neuroblasts [32] and adjacent to cilia in mature neurons [33].Fig. 1

This proximity is potentially functional, as mitochondria positioned at the ciliary base could supply ATP for energy-intensive processes such as IFT and Ca^2+^-dependent signaling [28]. Concurrently, ciliary activity can influence mitochondrial dynamics and bioenergetics, creating bidirectional metabolic coupling. Coordinating organelle position with local energy demand may facilitate cycles of cilium assembly/disassembly and adaptive mitochondrial remodeling, supporting cellular responses to stress. Together, these observations underscore the ciliary-mitochondrial axis as a contributor to spatiotemporal metabolic regulation with implications for neuroprotection and disease.

### Ciliary regulation of mitochondrial homeostasis

Recent work shows that primary cilia play a central role in controlling mitochondrial morphology and bioenergetics. For example, the BBSome complex regulates phosphorylation and recruitment of the mitochondrial fission regulator dynamin-like protein 1 (DRP1) [34, 35]. Balanced mitochondrial fission and fusion are essential for organelle quality control, metabolic flexibility, and neuronal health, and disruption of this balance impairs neuronal responses to metabolic stress and contributes to neurodegeneration [36]. Notably, loss of BBSome function causes excessive mitochondrial elongation and reduced mitochondrial function, while depletion of IFT88 lowers oxidative phosphorylation and fatty acid oxidation [37]. Together, these findings indicate that compromised ciliary integrity perturbs mitochondrial dynamics and energy production, leading to widespread cellular dysfunction.

Primary cilia also directly influence mitochondrial metabolism via Ca^2+^-dependent signaling. The ciliary membrane contains Ca^2+^ channels that activate the mitochondrial ATP-Mg/Pi solute carrier 25A25 (SLC25A25), linking ciliary sensing to bioenergetic adaptation [30]. Similarly, ciliary membrane proteins polycystin-1 (PC1) and polycystin-2 (PC2) modulate intracellular Ca^2+^ flux and thereby affect mitochondrial positioning and activity [38, 39]. Together, these molecular interactions establish a ciliary-mitochondrial signaling axis that fine-tunes energy balance and supports metabolic resilience.

### Mitochondria maintain primary cilia health

Mitochondrial bioenergetic function preserves primary cilia integrity. For example, genome-wide RNA interference (RNAi) screens have identified respiratory chain components as key modulators of ciliary disassembly, with mitochondrial gene perturbations disrupting this process [40]. Several proteins classically associated with mitochondria also localize to the ciliary basal body and directly regulate ciliogenesis and continuous cycles of assembly and disassembly to preserve sensory and signaling function, including the voltage-dependent anion channels (VDAC1–3). These channels, which are canonically linked to mitochondrial outer membrane permeability, and also localized to centrosomes where they exert opposing effects, with VDAC1 and VDAC3 driving ciliary disassembly and VDAC2 promoting ciliary growth [41]. Mitochondrial dynamics likewise shape cilia architecture, with loss of the mitochondrial fusion protein optic atrophy 1 (OPA1) leading to ciliary elongation, and DRP1 depletion shortening cilia [42]. Together, these findings position mitochondria as metabolic guards that tune ciliary structure and function, highlighting the ciliary-mitochondrial axis as a bidirectional regulatory circuit. Understanding these interdependencies could reveal new ways to modulate organelle crosstalk and preserve neuroprotective neuronal homeostasis.

### Pathological insights from ciliopathies and mitochondriopathies

Studies of ciliopathies, a heterogenous group of disorders caused by mutations in genes that govern the ciliumcentrosome complex, have provided insights into the functional linkage between cilia and mitochondria. Since the late twentieth century, genes implicated in ciliopathies have consistently been associated with mitochondrial defects, including impaired bioenergetics, increased oxidative stress, and disrupted mitochondrial quality control (Table 1). Notably, mitochondriopathies often show ciliary structural abnormalities. This bidirectional relationship indicates a shared molecular nexus in which dysfunction in one organelle worsens the other, accelerating disease. Importantly, recent preclinical work shows that restoring mitochondrial function can rescue ciliary defects in ciliopathy models, suggesting mitochondrial pathways as promising therapeutic targets [48, 54]. These findings support the ciliary-mitochondrial axis as both a driver of disease and a potential point of intervention to restore cellular homeostasis.

## Aging-associated dysregulation of the ciliary-mitochondrial axis

**Fig. 2:**
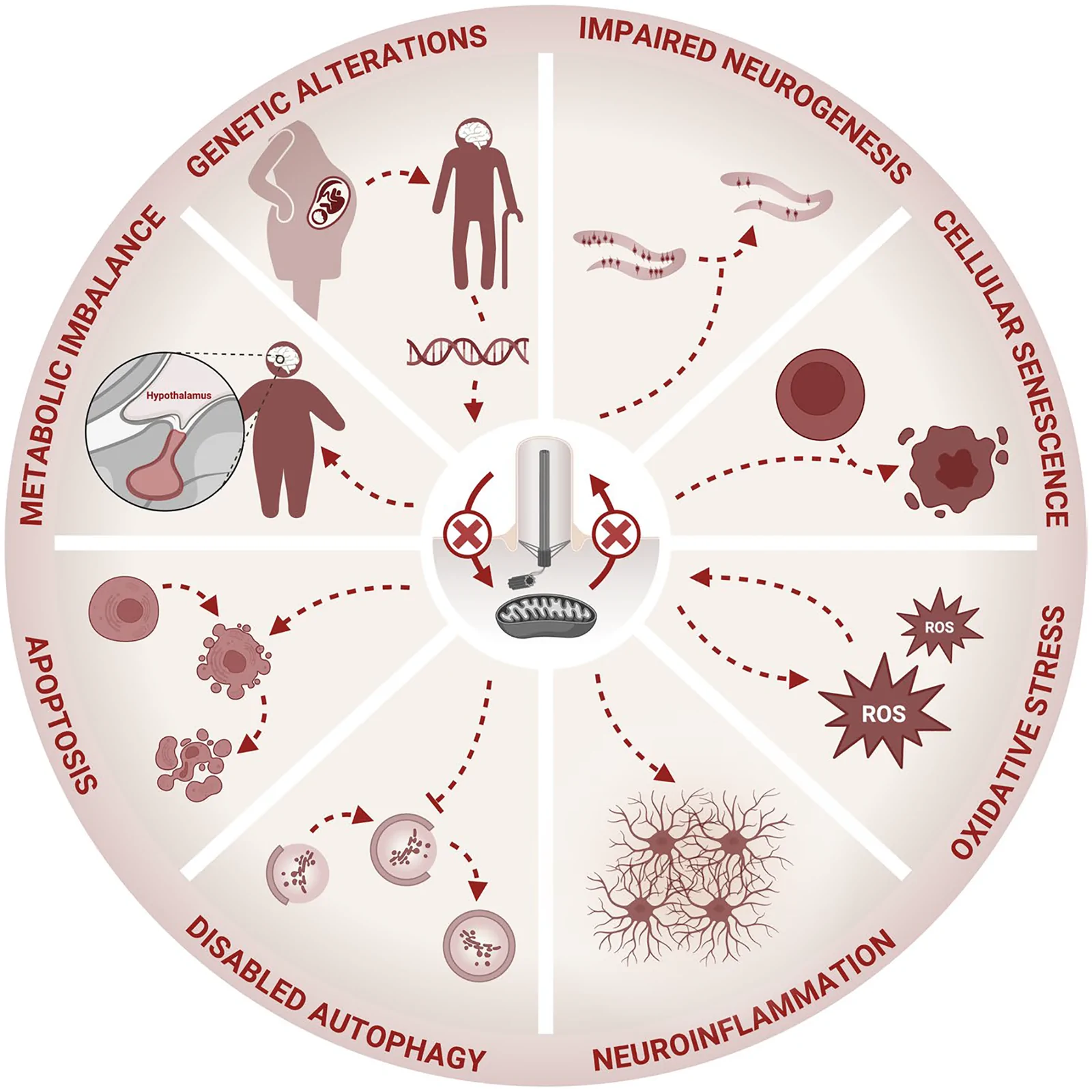
Deterioration of the Primary Cilia-Mitochondrial Axis in Aging. Progressive decline in the primary cilia-mitochondrial axis is associated with pathophysiological features of aging. Deterioration of the inter-organellar mechanisms leads to a breakdown in cellular function and resilience, underscoring the importance of the cilia-mitochondrial axis in maintaining cellular health during aging

Aging commonly causes progressive decline in neurological and cognitive function. Thus, identifying mechanisms that support lifelong resilience is important. The ciliary-mitochondrial axis has emerged as a guardian of brain health, with its functional integrity promoting neuroprotective adaptations and its dysregulation contributing to age-related impairments in metabolism, genetic regulation, postnatal neurogenesis, neuroplasticity, cellular senescence, oxidative stress control, neuroinflammation, autophagy, apoptosis, and development of neurodegenerative disease (Fig. 2).

### The ciliary-mitochondria axis in metabolism

Obesity, an age-exacerbated global metabolic health crisis, overlaps strikingly with ciliopathies, which often feature abnormal weight gain as hypothalamic cilia lose function. The ciliary-mitochondrial axis is central to metabolic homeostasis, as evidenced by age-related deterioration of hypothalamic primary cilia driving adiposity, leptin resistance, and impaired sympathetic tone [55]. Preclinical studies show obesity-associated ciliary loss in hypothalamic nuclei that regulate feeding, including the arcuate nucleus and paraventricular nucleus, that coincides with mitochondrial dysfunction and disrupted energy balance [56–60]. For example, loss of cilia in ventromedial hypothalamic neurons impairs mitochondrial integrity, and deletion of IFT88 reduces both ciliary structure and mitochondrial DNA (mtDNA) content. These disturbances reprogram cellular metabolism toward lipid storage and away from thermogenic energy expenditure [61]. Together, these data frame the ciliary-mitochondrial axis as a metabolic rheostat whose age-related decline may drive systemic energy imbalance, thereby serving as a target for restoring metabolic health in aging.

### The ciliary-mitochondria axis in genetic regulation

Recent studies demonstrate that mitochondria play a critical role in regulating ciliary gene expression and structural integrity in neurons [62]. In striatal D1-type medium spiny neurons, mitochondrial activity operates upstream of ciliary function, influencing ciliary length and assembly and thereby modulating transcriptional programs essential for neuronal signaling. Disruption of mitochondrial dynamics, particularly downregulation of mitofusin 2 (Mfn2), reduces expression of key ciliary genes (for example, Crocc/rootletin) and produces shortened, structurally altered neuronal cilia [62]. These results indicate that mitochondrial integrity is a fundamental determinant of ciliary morphology via transcriptional regulation, highlighting an integrated organelle network that shapes neuronal function. Notably, restoration of ciliary components can correct structural deficits without inducing apoptosis in neurons [62], supporting the ciliary–mitochondrial axis as a potential therapeutic target in neurological disorders.

Complementary work in astrocytes shows that mitochondrial dysfunction directly reprograms ciliogenesis [63]. For example, mtDNA depletion, an established marker of aging, induces aberrant ciliogenesis by impairing oxidative phosphorylation and activating transcriptional regulators such as Forkhead Box J1 and Regulatory Factor X [63]. This reprogramming disrupts astrocyte homeostasis and promotes formation of reactive astrocyte phenotypes. Together, these observations implicate mitochondria as upstream regulators of ciliary homeostasis in both neurons and glia.

Lastly, large-scale transcriptomic analysis from the BrainSpan Atlas further indicates that primary cilia in the human brain are increasingly vulnerable to age-related deterioration. Aberrations are observed across multiple ciliary components, including basal bodies, GPCRs, IFT proteins, transition fibers, and axonemes, each of which can impair ciliogenesis and ciliary function [64]. These deficits are most pronounced in the ventrolateral prefrontal cortex, posterior superior temporal cortex, and primary visual cortex, regions integral to memory, decision-making, emotional regulation, language, auditory processing, and visual perception. Recognizing vulnerability of the ciliary–mitochondrial axis in aging suggests new avenues for interventions aimed at preserving organelle cross-talk as a strategy to protect brain function in aging.

### The ciliary-mitochondria axis in postnatal neurogenesis and neuroplasticity

Over the past two decades, primary cilia have been established as major regulators of postnatal neurogenesis, synaptic plasticity, and neural circuit integrity, processes that underlie lifelong neuroplasticity [65–70]. Anchored at the centrosome, cilia function as signaling hubs that coordinate neurogenic programs and maintain adaptive connectivity across the lifespan [71, 72]. Sonic hedgehog (Shh) signaling, localized to primary cilia, promotes neurogenesis and plasticity and is disrupted in age-related cognitive decline [73]. Recent work links Shh activity to mitochondrial modeling, with Shh-driven suppression of Drp1-mediated mitochondrial fission leading to elongation, increased oxidative phosphorylation, and enhanced axonal growth in hippocampal neurons [74]. Similarly, engrailed-1 (EN1) modulates the ciliary-mitochondrial axis by tuning ciliary Wnt signaling to protect mitochondrial complex I activity and bioenergetic balance in dopaminergic neurons [75]. Together, these findings position primary cilia as metabolic integrators that synchronize mitochondrial adaptations with neuroplastic demands, while mitochondrial feedback preserves ciliary signaling. This bidirectional circuit supports neuronal resilience with aging and identifies targets for therapies to restore plasticity in neurodegenerative and age-related disorders.

### The ciliary-mitochondria axis in cellular senescence

Primary cilia coordinate cell cycle progression in quiescent cells and are disassembled when cells re-enter the cycle. Cellular senescence, an evolutionarily conserved irreversible cell cycle arrest triggered by stress, protects against cancer and accumulates with age, impairing tissue homeostasis and increasing neurodegeneration [76]. Dysregulation of the ciliary-mitochondrial axis is a key contributor to pathological functioning of this process.

NIMA-related kinase 4 (NEK4) stabilizes the primary cilium at its base and activates Drp1 to promote mitochondrial fission [77]. NEK4 loss disrupts ciliary structure [78], causes mitochondrial elongation, and produces oxidative respiratory defects, which collectively favor senescence. Conversely, inhibition of aurora kinase A (AURKA), which normally promotes ciliary disassembly and maintains cell cycle progression [79], also triggers mitochondrial elongation and functional decline, mirroring the dysfunction observed in NEK4-depleted systems [80]. Thus, both excessive ciliary disassembly (via NEK4 loss) and excessive ciliary stabilization (via AURAK deficiency) perturb mitochondrial homeostasis and cell-cycle coordination. These findings indicate that senescence arises not from static ciliary states, but from disrupted equilibrium between ciliary dynamics and mitochondrial fidelity, underscoring the necessity of axis balance for cellular vitality. Restoring this equilibrium might mitigate age-related senescence.

### The ciliary-mitochondria axis in oxidative stress control

Aging is characterized by increased oxidative damage to lipids, proteins, and DNA, in large part due to reactive oxygen species (ROS) that are generated as natural by-products of mitochondrial metabolism. The effects of ROS also link mitochondrial dynamics and ciliary remodeling in a bidirectional feedback loop. Stress-induced loss of heat shock protein family A member 9 (HSPA9) promotes mitochondrial fission and ROS accumulation [81], while activation of the energy sensor adenosine monophosphate-activated protein kinase (AMPK) stimulates ciliogenesis [42]. Together, these responses couple mitochondrial fragmentation to compensatory ciliary growth, with AMPK-driven ciliogenesis in turn activating neuroprotective AKT signaling, suggesting that ciliary expansion may provide an adaptive survival mechanism that counteracts mitochondrial dysfunction. Antioxidant signaling via nuclear factor-erythroid 2-like 2 (Nrf2) exhibits context-dependent effects on ciliogenesis, either promoting or inhibiting it [82, 83], a duality with currently unclear mechanisms. Overall, ROS functions as a key modulator of the ciliary-mitochondrial axis and deeper understanding this cross-talk could enable targeted interventions that reduce age-related oxidative damage and neurodegeneration.

### The ciliary-mitochondria axis in neuroinflammation

Acute immune signaling can be neuroprotective, but chronic low-grade neuroinflammation in aging increases the risk of neurodegeneration. The ciliary-mitochondrial axis acts as a rheostat that balances pro- and anti-inflammatory responses. For example, mtDNA depletion induces ciliary elongation in astrocytes, which adopt a neurotoxic reactive state characterized by increased cytokine release and complement C3 activation [63]. Targeted ablation of astrocytic cilia attenuates neurotoxic inflammation [84]. Conversely, pathogenic loss or shortening of cilia can suppress pro-inflammatory pathways such as nuclear factor kappa B (NF-κB), which are coupled to mitochondrial biogenesis, ROS homeostasis, and metabolic adaptation [85]. Thus, transient ciliary loss may initially dampen inflammation, but chronic ciliary dysfunction disrupts ciliary-mitochondrial feedback and can worsen pathology. Understanding these context-dependent effects could identify interventions that reduce chronic neuroinflammation while preserving neuroprotective signaling.

### The ciliary-mitochondria axis in autophagy

Autophagy, a key neuroprotective process for recycling damaged cellular components, declines with age and is impaired in ciliopathies, where mutations in cilia-related genes reduce autophagic flux [86–88]. Primary cilia regulate autophagy, with activity suppressed by cilia shortening and stimulated by ciliary elongation [89]. Cilia also act as scaffolds for autophagy machinery, concentrating proteins such as ATG16L, AMBRA1, LC3, GABARAP and VPS15 [90]. IFT and ADP-ribosylation factor-like 3 (ARL3) maintain ciliary structure and ensure proper localization of these autophagy components, while disruption of IFT or ARL3 impairs autophagosome formation and autophagy [91].

Mitochondrial stress exacerbates this interdependence via ROS-AMPK signaling, which synchronizes ciliogenesis and autophagy to promote mitophagy and cellular survival [42]. Thus, primary cilia integrate mitochondrial stress signals to recruit autophagic machinery and optimize degradation. Loss of this coordination impairs stress adaptation and increases cell death, suggesting that restoration of ciliary-mitochondrial coupling could help preserve proteostasis in aging.

### The ciliary-mitochondria axis in apoptosis

Apoptosis is a tightly regulated form of programmed cell death that preserves tissue integrity by removing damaged cells, but becomes harmful when dysregulated in injury or neurodegeneration. Loss of ciliary structure or function promotes neuronal apoptosis, while ciliary stabilization protects neurons from inappropriate cell cycle re-entry and downstream apoptotic signaling [92–94]. Mechanistically, ciliary loss can trigger mitochondria-dependent apoptosis, as exemplified by induction of VDAC1 oligomerization in response to IFT downregulation, a hallmark of mitochondrial permeability transition and caspase activation [95]. Reduced expression of ciliary ARL3 expression also impairs mitochondrial bioenergetics and leads to apoptotic cell death [96]. Conversely, mitochondrial stress, such as inhibition of the respiratory chain or excessive mitochondrial fission, can induce compensatory ciliogenesis that helps neurons evade apoptosis [42]. Thus, therapeutic strategies that restore the balance of the ciliary-mitochondrial axis may reduce apoptosis-driven pathologies.

### The ciliary-mitochondria axis in development of neurodegenerative disease

Ciliopathies, traditionally viewed as developmental disorders, share many phenotypes with neurodegenerative diseases, including cognitive decline, brain atrophy, anosmia, retinal degeneration, and insomnia [97–99]. Increasing evidence implicates adult-onset ciliary dysfunction in age-related neurodegeneration, with genomic studies reporting dysregulation of ciliary genes in human Alzheimer’s disease (AD) [100, 101] and preclinical models of Parkinson’s disease (PD) [102]. It is also notable that the ciliopathy polycystic kidney disease places individuals at a higher risk for dementia [103].

**Fig. 3:**
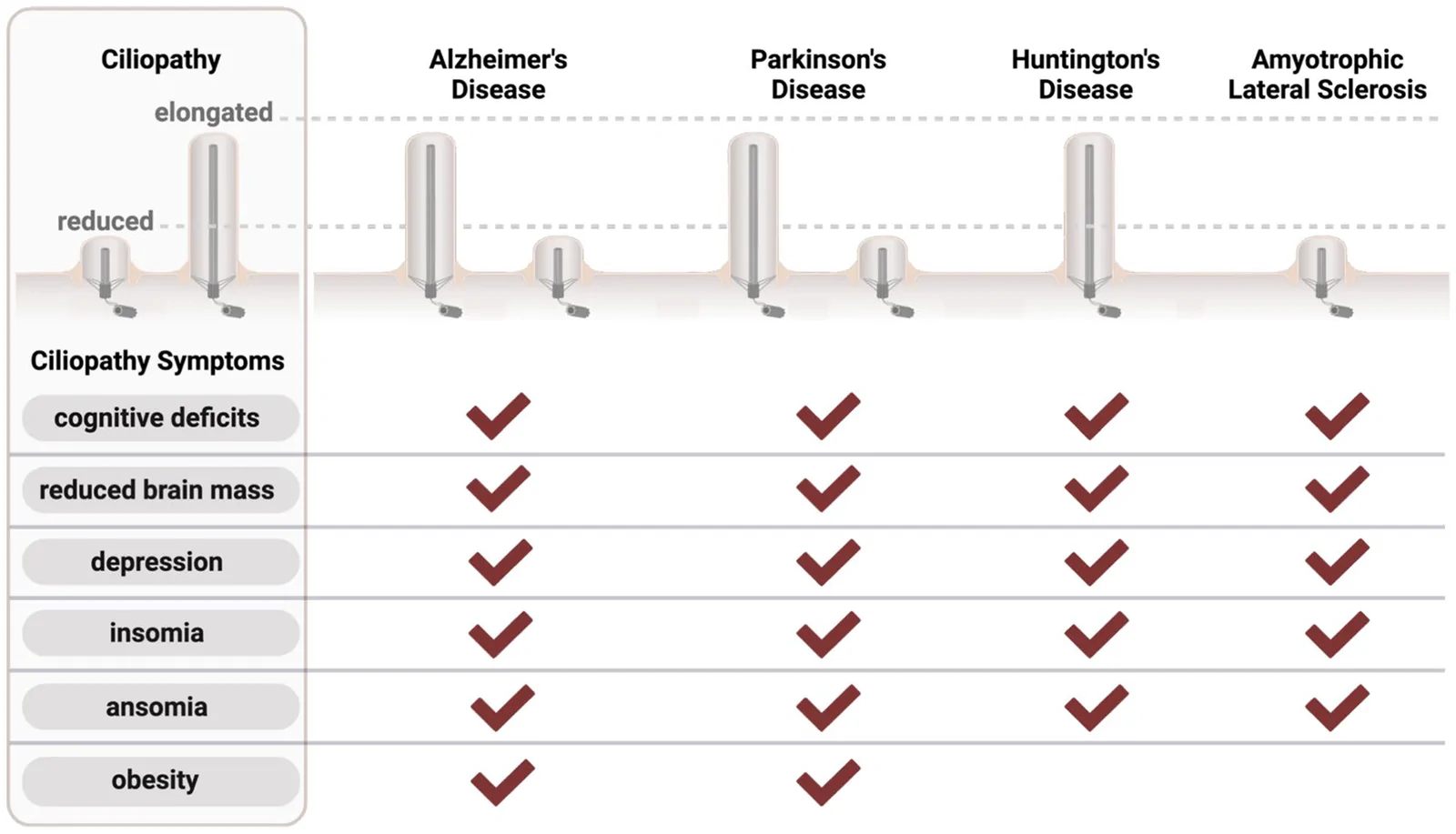
Ciliopathy Symptoms in age-related neurodegenerative diseases that are also Associated with mitochondrial pathology. Ciliopathies share clinical symptoms with major neurodegenerative disorders of aging, including Alzheimer’s disease [106–108], Parkinson’s disease [109–113], Huntington’s disease [114–117], and amyotrophic lateral sclerosis [118–121]. These conditions are also associated with impaired mitochondrial function

Although whether adult ciliary defects are a primary cause of neurodegeneration is still debated, preclinical models show that cilia loss accelerates neurodegeneration. For example, loss of tau tubulin kinase 2 (TTBK2) prevents ciliogenesis and induces neuronal death [104], and NEKL-4 deficiency impairs ciliary kinesin activation and axoneme stability, leading to CCP1-mediated neurotoxicity through mitochondrial dysfunction [105]. These studies indicate that disruption of the ciliary-mitochondrial axis drives neurodegeneration rather than merely correlating with it. Given the well-established role of mitochondrial dysfunction in neurodegenerative diseases, the ciliary-mitochondrial axis emerges as a shared mechanistic nexus across multiple disorders (Fig. 3). Understanding how defective ciliary signaling converges on mitochondrial failure could reveal new therapeutic strategies to halt or reverse neurodegeneration.

### The ciliary-mitochondrial axis in alzheimer’s disease

While the role of mitochondria in AD has been extensively characterized, cilia have been less well studied in this condition. Recently, however, whole genome sequencing in AD patients identified mutations linked to ciliary dysfunction, notably in genes required for basal body function and plasma membrane docking [101]. Proteomic analyses likewise show overlap between ciliary and AD-associated protein networks, with enrichment for neurogenesis-related genes and druggable targets [100]. Furthermore, ciliary alterations have been reported in multiple mouse AD models. 3xTg AD mice, for example, exhibit reduced levels of cilia-enriched receptors critical for neuronal proliferation and maturation, suggesting possible cilia shortening in AD [122]. The APP/PS1 model also implicates the serotonin receptor 5-HT6 in cilia length regulation during disease progression [123], while genetic ablation of cilia in 5xFAD mice correlates with increased amyloid plaque burden [124]. Notably, amyloid precursor protein (APP) is enriched in primary cilia, modulates cilia length by unknown mechanisms [125–128], and has been proposed to act as an Aβ-sensing receptor in cilia, potentially perturbing Shh signaling and neuronal survival [126]. Overall, primary cilia dysfunction reproduces key AD phenotypes, including cortical and hippocampal atrophy, ventricular enlargement, and cognitive deficits [97–99, 104, 129, 130]. Given the clear role of both cilia and mitochondria in the pathophysiology of AD, future study of the ciliary-mitochondrial axis in AD is a promising area for therapeutic discovery.

### The ciliary-mitochondrial axis in parkinson’s disease

Single-cell transcriptomics of hiPSC-derived neural precursor cells from people with PD also implicate primary cilia dysfunction [102]. Specifically, genes encoding IFT and BBSome components are dysregulated, suggesting impaired receptor trafficking in cilia. Transcriptomic and proteomic profiling of CD271 + cells from the subventricular zone of post-mortem PD brains supports this, showing downregulation of genes required for ciliogenesis and ciliary function [131]. Functionally, loss of cilia in D1 dopamine receptor-expressing neurons produces locomotion deficits, a core PD symptom [132]. Several familial PD genes also regulate ciliogenesis. Mutations in leucine-rich repeat kinase 2 (*LRRK2*) for example, disrupt early cilia formation by blocking TTBK2 recruitment, preventing CP110 removal from centrioles and blocking axoneme elongation [133]. Pathogenic LRRK2 kinase activity further blocks ciliogenesis via Rab10 phosphorylation and RILPL1 binding, suppressing ciliary Shh signaling [134], a pathway vital for dopaminergic neuron resilience to toxins [135]. Mutations in *PINK1* and engrailed-1 (*EN1*) likewise impair ciliogenesis and mitochondrial function, promoting dopaminergic neuron loss and motor decline [136–138]. Together, these studies emphasize the interdependence of ciliary and mitochondrial integrity for dopaminergic neuron survival and point to the ciliary-mitochondrial axis as a potential source for new therapeutic targets in PD.

### The ciliary-mitochondria axis in psychiatric conditions

Growing evidence also links primary cilia dysfunction to major neuropsychiatric disorders, including schizophrenia (SZ), bipolar disorder (BD), autism spectrum disorder (ASD), and major depressive disorder (MDD) [100, 139–141]. A central example is disrupted in schizophrenia 1 (DISC1) protein, which connects ciliary and mitochondrial pathology in psychiatric illness. DISC1 regulates primary cilia formation and dopamine receptor localization in both SZ and BD [140, 142], and its dysfunction perturbs cilia-dependent Shh and Wnt signaling pathways essential for neuronal development and maintenance [143–145]. DISC1 also controls mitochondrial trafficking via Miro1-GTPase, coupling ciliary defects to bioenergetic impairment in SZ and BD [146]. In preclinical models, DISC1 deficiency impairs mitochondrial axonal transport, promoting neurodegeneration and cognitive deficits that are reversible with DISC1 restoration [147].

### Therapeutic modulation of the ciliary-mitochondrial axis

As detailed above, primary cilia and mitochondria are tightly interconnected despite their structural and functional differences. Mitochondria influence ciliary gene expression [62], while ciliogenesis and ciliary signaling depend on mitochondrial proteins and bioenergetics [28, 48]. Ciliary calcium flux modulates mitochondrial activity, producing bidirectional communication in which dysfunction of one organelle propagates to the other. Thus, therapeutic interventions targeting the ciliary-mitochondrial axis may be particularly effective.

Nicotinamide adenine dinucleotide (NAD^+^) exemplifies the therapeutic potential of this axis. As a central coenzyme for mitochondrial metabolism, NAD^+^ supports energy production, DNA repair, and redox balance. Age-related NAD^+^ decline worsens mitochondrial dysfunction and ciliary defects in neurodegeneration and ciliopathies. In models of Joubert syndrome harboring mutations in the basal body protein ARMC9, for example, NAD^+^ supplementation restores ciliogenesis by promoting ARMC9 interaction with the mitochondrial assembly factor NADH ubiquinone oxidoreductase complex assembly factor 2 (NDUFAF2), enabling clearance of CP110, a ciliogenesis inhibitor, while simultaneously reversing mitochondrial bioenergetic deficits [48]. By contrast, diseases characterized by ciliary-mitochondrial axis hyperactivity, such as polycystic liver disease, can benefit from NAD^+^ suppression. For example, pharmacological inhibition of NAD^+^ synthesis via FK866 attenuates mitochondrial overactivity and aberrant cyst proliferation [44].

These outcomes reveal the axis’s role as a tunable rheostat for cellular homeostasis. Modulating NAD^+^ according to disease-specific imbalances can recalibrate organelle communication for therapeutic benefit. Future therapies will likely require coordinated targeting of both organelles. Strategies that concurrently restore mitochondrial function and ciliary signaling, and that exploit regulatory nodes across mitochondrial stress responses and ciliogenesis, may yield effective precision treatments for aging-related disorders, including neurodegenerative disease.

## Concluding remarks: the ciliary-mitochondrial axis as a guardian of neuronal resilience

The ciliary-mitochondrial axis is a central regulatory hub that integrates sensory, metabolic, and stress-adaptive signaling to maintain neuronal health. This bidirectional relationship controls processes vital to aging and neurodegeneration, including metabolic equilibrium, postnatal neurogenesis, synaptic plasticity, redox balance, and proteostasis, with dysfunction in either organelle perturbing the whole system. Age-related decline of the axis produces a pathogenic feedback loop in which mitochondrial failure and ciliary dysfunction mutually reinforce one another, synergistically promoting senescence, neuroinflammation, and neuronal loss.

Importantly, these interdependent processes are modifiable. Preclinical studies show that targeted reinforcement of the axis can reverse degenerative cascades, highlighting its dual role as both a mechanism of neuronal resilience and a tractable therapeutic target. Future work should prioritize both mechanistic dissection of context-specific regulators that tune ciliary-mitochondrial crosstalk and development of precision therapies to restore axis dynamics. Framing organellar miscommunication in the ciliary-mitochondrial axis as a causal contributor to aging and neurodegeneration opens a new therapeutic paradigm for preserving brain health across the lifespan.

## Figures and Tables

**Table 1:** Ciliopathies and mitochondriopathies: effects on primary cilia and mitochondrial health

Disease	Protein Deficiency	Impact on Primary Cilia/Mitochondria	Related Mechanism	Refs
**Ciliopathies**				
Autosomal dominant polycystic kidney disease (ADPKD)	PKD1 or PKD2	Reduced mitochondrial DNA, fragmented mitochondria	Mutations disrupt polycystin calcium channels in primary cilia, causing oxidative stress, ROS overproduction, and downregulation of PGC1α (impairing mitochondrial biogenesis)	[38,43]
Polycystic liver disease (PLD)	NAMPT overexpression	Hyperactive mitochondrial oxygen consumption and ATP production	Ciliary dysfunction drives NAMPT/NAD + signaling. This is known to activate SIRT1/3 and PGC-1α to induce mitochondrial hyperactivity	[44,45]
Nephronophthisis	XPNPEP3 (NPHPL1)	Defects in mitochondrial Complex I, elongated cilia	XPNPEP3 stabilizes mitochondrial Complex I and regulates cilia length. Mutations impair oxidative phosphorylation (↓ ATP), increase ROS, and trigger apoptosis	[46]
Bardet-Biedl syndrome (BBS)	BBSome complex (e.g., BBS10, BBS1)	BBS1: elongated mitochondria, reduced OCR, reduced calcium handling BBS10: Reduced mitochondrial membrane potential, decreased citrate synthase activity, and accumulation of lipid droplets in renal tubular cells, reduced primary cilia length	Mutations in BBSome components disrupt mitochondrial dynamics and ciliary function. BBS1 phosphorylates and translocate DRP1 to mitochondria, where it promotes mitochondrial fission and calcium signaling. BBS10 deficiency impairs the PINK1/Parkin-mediated mitophagy pathway which leads to the accumulation of dysfunctional mitochondria, and renal dysfunction	[34,47]
Joubert Syndrome	ARMC9	Increased prevalence of onion-shaped cristae and a reduced number of cristae, coupled with a higher abundance of filamentous mitochondria, elevated oxidative stress levels, shorter cilia length	Mutations in the basal body protein ARMC9 lead to reduced levels of the intrinsic mitochondrial complex I component NDUFAF2, resulting in impaired mitochondrial activity and elevated oxidative stress	[48]
Jeune asphyxiating thoracic dystrophy (JATD)	IFT80	Abnormal mitochondrial structure, shorter cilia and reduced ciliary frequency	Mutations in IFT80 disrupt ciliary transport, unknown mechanisms related to impaired mitochondrial integrity	[49]
Alstrom syndrome	ALMS1	Slight mitochondrial pleomorphism, increased oxidative stress, mitochondrial dysfunction, shorter cilia	ALMS1 mutations cause ciliary dysfunction, unknown mechanisms related to mitochondrial stress	[50,51]
**Mitochondriopathies**				
Leigh Syndrome	NDUFAF2	Impaired ciliogenesis and mitochondrial dysfunction, reduced complex I activity and increased oxidative stress and mitochondrial DNA deletion	NDUFAF2 regulates ciliogenesis by interacting with ARMC9 and removing CP110, linking mitochondrial defects to ciliary abnormalities	[48]
Barth Syndrome	TAZ	Reduced primary cilia formation	TAZ mutations disrupt cardiolipin remodeling, inhibiting ciliogenesis	[52]
Mitochondrial DNA depletion syndrome (MDDS)	MPV17	Increased cilia length and impaired energy production	Mutations in MPV17 reduce mitochondrial DNA and affect ciliogenesis, linking mitochondrial respiration to ciliary dynamics	[53]

## Abbreviations

5-HT6: 5-Hydroxytryptamine 6 receptor
Aβ: Amyloid beta
ADP: Adenosine diphosphate
ADPKD: Autosomal dominant polycystic kidney disease
AD: Alzheimer’s disease
Akt: Protein kinase B
ALMS1: Alstrom syndrome 1
AMBRA1: Activating molecule in Beclin1-regulated autophagy
AMPK: Adenosine monophosphate-activated protein kinase
ARL3: ADP-ribosylation factor-like 3
ARMC9: LisH domain-containing protein
ASD: Autism spectrum disorder
ATG16L: Autophagy related 16 like 1
AURKA: Aurora kinase A
ATP: Adenosine triphosphate
BBSome: Bardet-Biedl Syndrome (BBS) proteins
BBS1: Bardet-Biedl syndrome 1
BBS10: Bardet-Biedl syndrome 10
BD: Bipolar disorder
Ca^2+^: Calcium ion
CCP1: Cytotoxic cell proteinase-1
CP110: Centriolar coiled-coil protein of 110 kDa
DISC1: Disrupted-in-schizophrenia 1
DNA: Deoxyribonucleic acid
DRP1: Dynamin-like protein 1
EN1: Engrailed-1
GABARAP: Gamma-aminobutyric acid receptor-associated protein
GPCRs: G protein coupled receptors
HSPA9: Heat shock protein family A member 9
IFT: Intraflagellar transport
IFT-A: Intraflagellar transport protein A
IFT-B: Intraflagellar transport protein B
IFT80: Intraflagellar transport protein 80 homolog
IFT88: Intraflagellar transport protein 88 homolog
JATD: Jeune asphyxiating thoracic dystrophy
kDa: Kilodalton
LC3: Microtubule-associated proteins 1A/1B light chain 3B
LRRK2: Leucine-rich repeat kinase 2
MDD: Major depressive disorder
MPV17: Protein MPV17
mtDNA: Mitochondrial DNA
NAD+: Nicotinamide adenine dinucleotide
NAMPT: Nicotinamide phosphoribosyltransferase
NDUFAF2: NADH:ubiquinone oxidoreductase complex assembly factor 2
NEK4: NIMA-related kinase 4
NF-κB: Nuclear factor kappa B
NPHPL1: Nephronophthisis-like nephropathy-1
Nrf2: Nuclear factor-erythroid 2-like 2
OCR: Oxygen consumption ratio
OPA1: Optic atrophy 1
PC1: Polycystin-1
PC12: Polycystin-2
PD: Parkinson’s disease
PGC1a: Peroxisome proliferator-activated receptor gamma coactivator 1-alpha
PINK1: PTEN-induced kinase 1
PINK2: PTEN-induced kinase 2
PKD1: Polycystin 1
PKD2: Polycystin 2
PLD: Polycystic liver disease
Rab10: Ras-related protein 10
RILPL1: Rab Interacting Lysosomal Protein Like 1
RNAi: RNA interference
ROS: Reactive oxygen species
Shh: Sonic hedgehog
SIRT1: Sirtuin 1
SIRT3: Sirtuin 3
SLC25A25: Solute carrier 25 member 25
S: Schizophrenia
TAZ: Tafazzin
TTBK2: Tau tubulin kinase 2
VDAC1: Voltage-dependent anion channel 1
VDAC2: Voltage-dependent anion channel 2
VDAC3: Voltage-dependent anion channel 3
VPS15: Vacuolar protein sorting 15
Wnt: Wingless and int-1
XPNPEP3: Xaa-pro aminopeptidase 3
